# Partial tetraplegic syndrome as a complication of a mobilizing/manipulating procedure of the cervical spine in a man with Forestier's disease: a case report

**DOI:** 10.1186/1752-1947-5-529

**Published:** 2011-10-27

**Authors:** Maximilian J Hartel, Ulrich Seidel, Lukas Iselin, Aristomenis K Exadaktylos, Lorin M Benneker

**Affiliations:** 1 Department of Trauma-, Hand-, and Reconstructive Surgery, University Medical Center Hamburg-Eppendorf, Martinistrasse 52, 20246 Hamburg, Germany; 2 Department of Orthopedic Surgery, Bern University Hospital, Inselspital, CH-3010 Bern, Switzerland; 3 Department of Emergency Medicine, Bern University Hospital, Inselspital, CH-3010 Bern, Switzerland

## Abstract

**Introduction:**

Even if performed by qualified physical therapists, spinal manipulation and mobilization can cause adverse events. This holds true particularly for the cervical spine. In light of the substantial risks, the benefits of cervical spine manipulation may be outweighed by the possibility of further injury.

**Case presentation:**

We present the case of a 56-year-old Caucasian man with Forestier's disease who went to see a physiotherapist to relieve his aching neck while on a holiday trip. Following the procedure, he was transferred to a local hospital with a partial tetraplegic syndrome due to a cervical 6/7 luxation fracture. Reportedly, the physiotherapist took neither a detailed history, nor adequate diagnostic measures.

**Conclusions:**

This case highlights the potentially dangerous complications associated with cervical spine mobilization/manipulation. If guidelines concerning cervical spine mobilization and manipulation practices had been followed, this adverse event could have been avoided.

## Introduction

Even if performed by qualified physical therapists, spinal manipulation and mobilization of the cervical spine in particular can cause severe adverse events. There has been doubt that the benefits of manipulation and mobilization at the cervical spine outweigh the risks linked to it [1-4]. Several potentially life-threatening complications following spinal manipulation have been reported [5-11].

Here, we describe an example of a severe non-vascular complication. The adverse event may likely have been avoided if the physical therapist had taken a careful patient history prior to the procedure, as our patient already knew about his underlying degenerative disease.

### Case presentation

We present the case of a 56-year-old Caucasian man with Forestier's disease also known as diffuse idiopathic skeletal hyperostosis (DISH). Forestier's disease is a common spinal enthesopathy that is mostly encountered in men older than 50 years [12]. A prevalence of 28% has been found in autopsy specimens [13]. DISH is more common in patients with diabetes and gout [14].

Our patient sought the services of a local physical therapist while on vacation to obtain massages and other treatments for his aching and stiff neck. According to our patient, the physiotherapist (board certified per our patient's report), was more forceful in his manipulating than our patient was used to. He reports having had severe neck pain prior to a short period of unconsciousness. After the procedure, he was unable to mobilize himself off the table. Prior to this incidence the physiotherapist had reportedly not known about our patient's Forestier's disease. He supposedly had not asked about underlying diseases nor had our patient remembered to tell him.

**Figure 1:**
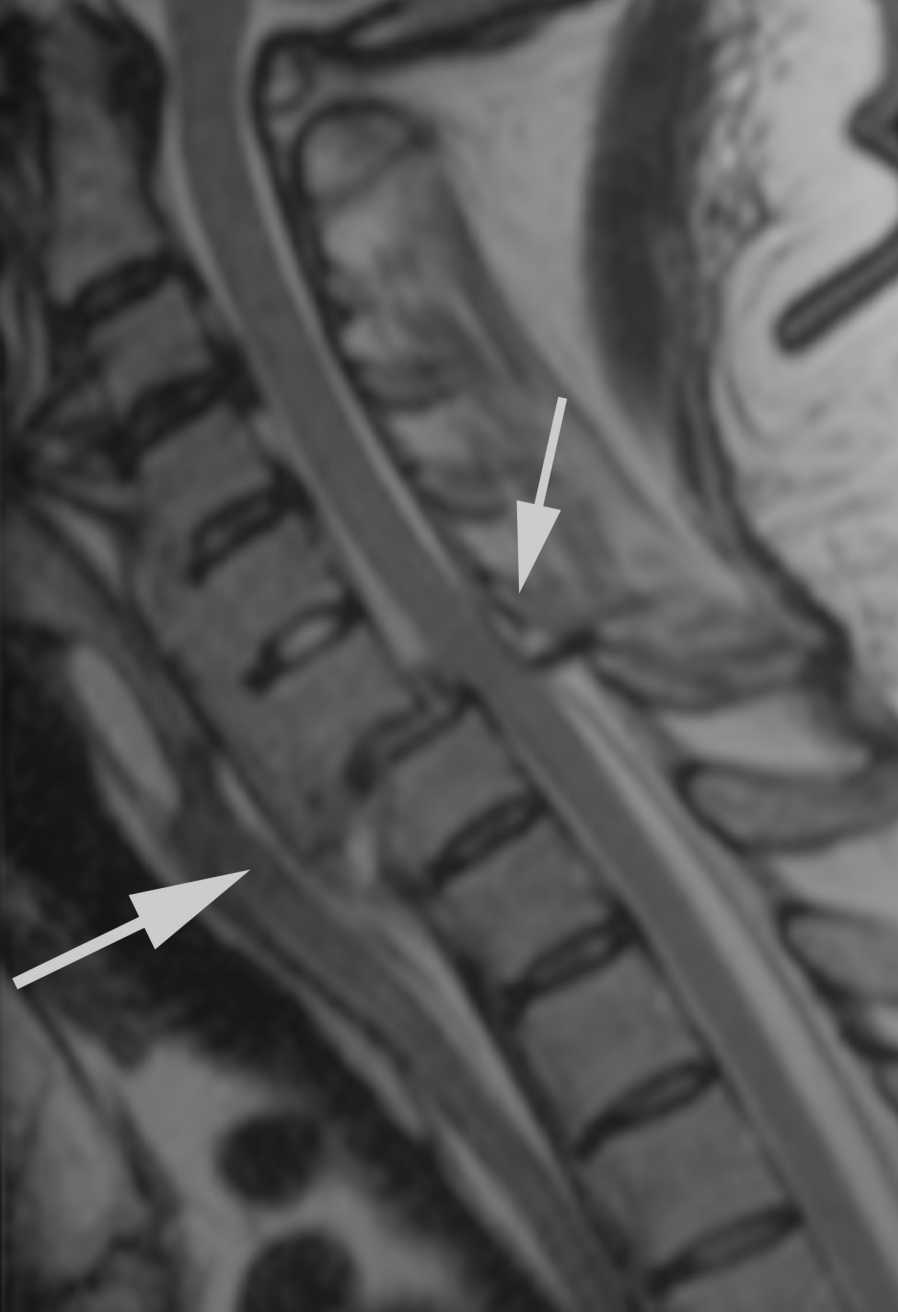
**T2-weighed MRI scan in a median-sagittal plane of the cervical spine**. There is a C 6/7 luxation fracture without evidence for a profound spinal cord lesion. The degenerative alterations, particularly the large bridging spondylophytes are consistent with Forestier's disease.

Our patient was referred to a local hospital with a partial tetraparetic syndrome. MRI scans of the cervical spine showed a C 6/7 luxation fracture, as well as degenerative alterations with large spondylophytes bridging the vertebral bodies of the cervical spine extensively (consistent with Forestier's disease) (Figure 1). Axial traction therapy was chosen for several days followed by a dorsal stabilization procedure using internal fixation. Twelve days after the initial trauma he was repatriated and referred to our division of spine surgery. Subjectively, the symptoms improved over time after the initial trauma. On admission to our institution our patient reported having electrifying pain in the whole left upper extremity. Finger abduction in the left hand was slightly reduced to grade M4 of five (according to British Medical Research Council grading, 1978). On the right side the active finger abduction was significantly reduced to grade M0-1/5 and elbow extension to M3/5.

**Figure 2:**
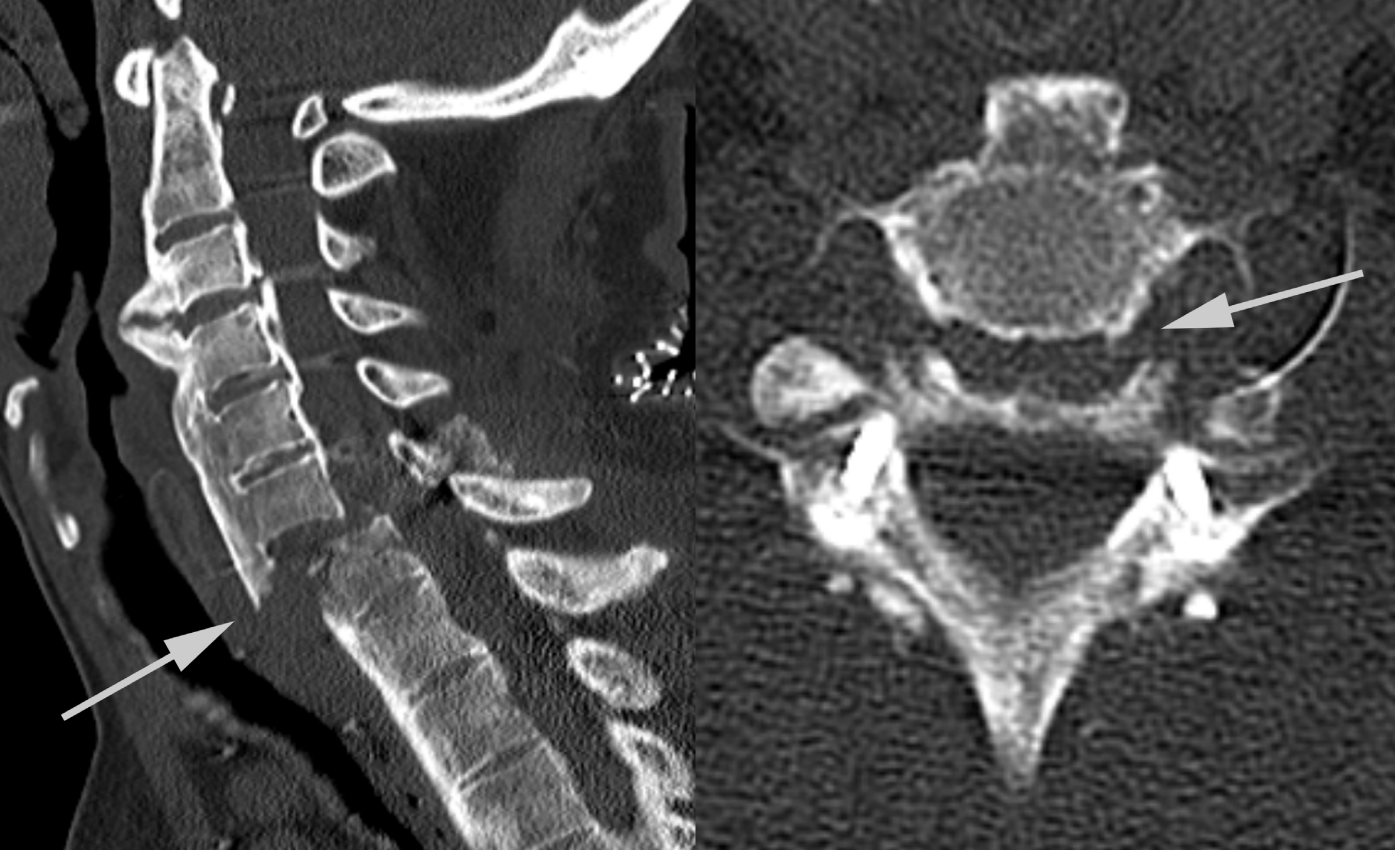
**Computed tomography scan of our patient's cervical spine obtained on admission to our institution**. The scan shows a persistent luxated position of the fracture at level C6/7 after dorsal stabilization at the outside hospital.

A subsequent computed tomography (CT) scan showed an insufficient fracture reduction leaving the facet joints in a persistent subluxation, potentially continuing to compromise neural structures (Figure 2). Our patient was also noted to have elevated inflammatory parameters. Due to his clinical presentation, revision surgery with posterior hardware removal, irrigation, debridement and decompressive laminectomy was undertaken. Our patient was then flipped into a supine position for a ventral approach. No obvious signs of infection were seen anteriorly and therefore a ventral inter-corporal fusion procedure was performed at level C6/7 and the cervical spine instrumented between the levels C5 to T1 using a plate (Vectra).

The results of cultures of the intra-operative biopsies taken from our patient's dorsal cervical spine were positive for a coagulase-negative *Staphylococcus *and *Proteus mirabilis*. An adequate antibiotic regime was established. Our patient was transferred to a neurological rehabilitation center eight days post-operatively in a stable condition.

**Figure 3:**
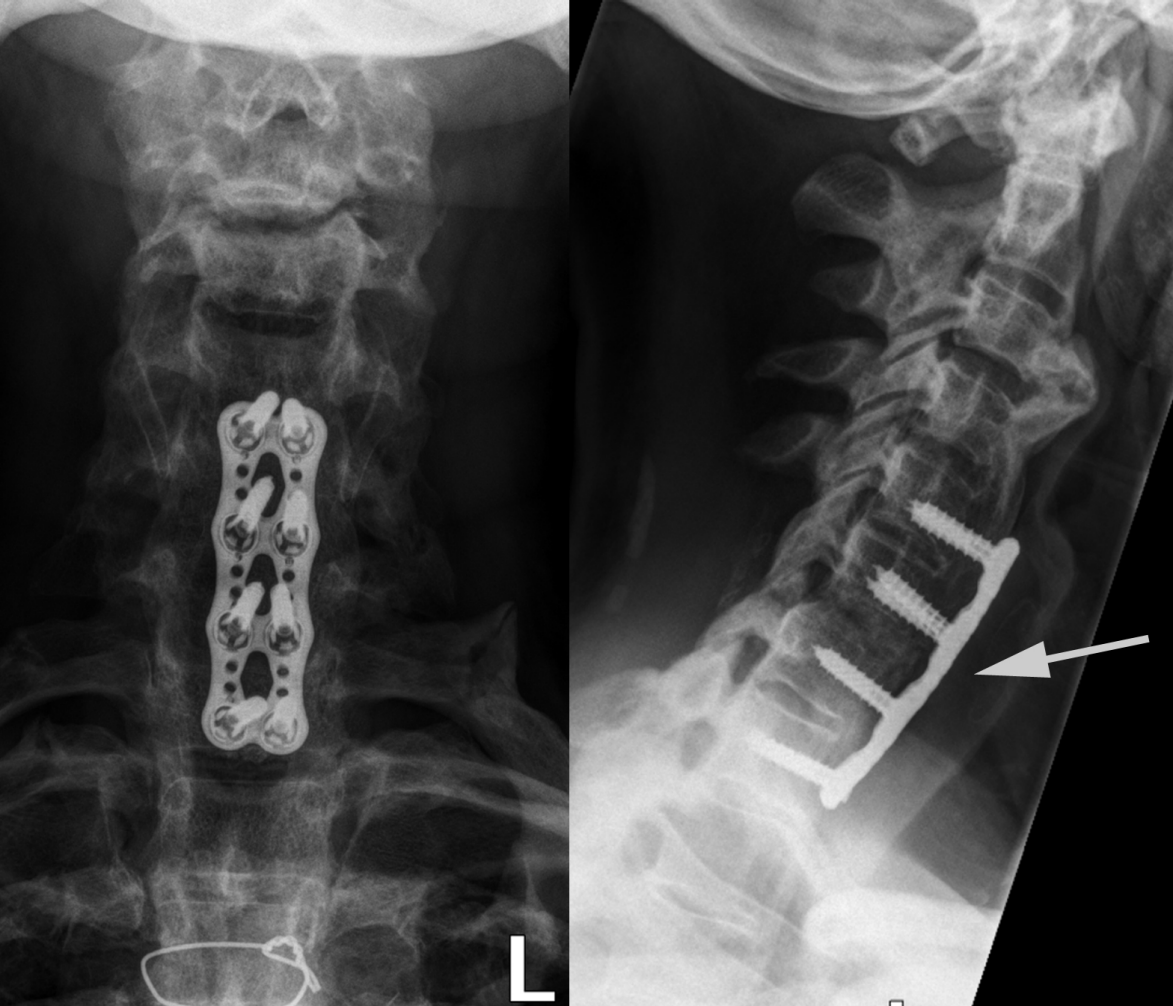
**Plain radiographic images obtained at the six-month follow-up**. The alignment is unchanged, the hardware intact, and there are signs of osseous consolidation.

At two-month follow-up, our patient reported satisfaction with the outcome. His inflammatory parameters had normalized and all the incisions looked well healed. He had an acceptable range of movement of his cervical spine. While his left upper extremity had full sensomotor function, on his right side function was still impaired. At six-month follow-up, persistent but slightly improving neurological deficits were recorded. Figure 3 shows radiographic imaging results obtained at the six-month follow-up demonstrating no changes in alignment, intact hardware and osseous consolidation. Subsequent to the last follow-up, our patient was still undergoing ergotherapeutic and physiotherapeutic therapy addressing his right upper extremity limitations.

## Discussion

Cervical manipulation and mobilization is commonly performed in cases of headache and neck pain [7]. Several potentially life-threatening complications following spinal manipulation have been reported [5-11]. Interestingly, there seems to be disagreement among experts in this field about the actual size of risk for complications following manual therapy procedures. Malone and colleagues estimated that in every 850 patients, one irreversible complication (for example, clinical significant vertebral disc herniations needing operative treatment) occurs [7]. Carnes *et al. *estimated in their review a very low risk rate of 0.01% per patient for major adverse events [15]. Then again, Kerry *et al. *state in their critical literature review in 2008 addressing the association between cervical spine manual therapy and cervical artery dysfunction, that 'it is currently impossible to meaningfully estimate the size of the risk of post-treatment complications' [16].

Fractures of the cervical spine seem to be a rare subgroup of the irreversible and serious complications associated with spinal manipulation. They are specifically reported in cases with pre-existing underlying spinal pathologies, such as osteoporosis, tumors or metastases [6,17,18]. Oppenheim *et al. *reported one pathological fracture in a series of 18 patients [5]. One case of a pathological odontoid fracture and another case of an osteoporotic odontoid fracture were seen by Schmitz *et al. *and Ea *et al*., respectively [6,19]. Kewalramani *et al. *reported two cases in their series of three [9]. In 1976, Rinsky and colleagues have published a case of a permanent C4 tetraplegia following chiropractic manipulation in a patient with ankylosing spondilitis [8]. To the best of our knowledge, the case presented in this paper is the first with Forestier's disease as the underlying pathology with a severe complication following a cervical mobilizing/manipulating procedure.

Our patient was aware of his underlying disease, but underestimated the risk of an adverse event and therefore neglected to inform his physiotherapist. As mentioned above, the complicated course of our patient may have likely been avoided if guidelines for treatment procedures involving the cervical spine had been followed [20,21]. Counter to standards of care, a detailed history was reportedly not taken by the provider [22]. Moreover, as postulated by Maigne *et al*., prior to any manipulation of the cervical spine radiographic imaging is indispensable [17]. Hurwitz and colleagues stated in their literature review that cervical spine mobilization and manipulation probably provide at least short-term benefit for some patients with neck pain or headaches [2]. Given this statement, mobilization and manipulation in the cervical spine may be justifiable for a limited number of patients [2,11].

If conventional X-ray imaging had been used in our patient's case, the underlying disease and the associated absolute contraindication for mobilization/manipulation practices would have been easily detected.

A limiting factor in this case is that our patient was not able to retrospectively depict the exact maneuver performed by the physiotherapist that led to the accident.

## Conclusions

The case serves as a reminder to health care providers of the potentially severe complications associated with cervical spine mobilization/manipulation. It emphasizes that cases such as this could easily be prevented if a thorough history had been taken and/or necessary diagnostic measures had been performed in advance of any mobilizing/manipulating procedure.

## Consent

Written informed consent was obtained from the patient for publication of this case report and any accompanying images. A copy of the written consent is available for review by the Editor-in-Chief of this journal.

## Competing interests

The authors declare that there are no competing interests that could inappropriately influence the content of this case presentation.

## Authors' contributions

LMB conceived the idea of the study. All authors helped to collect the data included in this case presentation. MJH, US and LMB were directly involved in the care of our patient. MJH was involved in the conception of the report, literature review, manuscript preparation, editing and submission. All authors read and contributed to the editing and review of the manuscript and gave their approval for the final manuscript.
